# Comparative phylogeography of *Aedes* mosquitoes and the role of past climatic change for evolution within Africa

**DOI:** 10.1002/ece3.3668

**Published:** 2018-02-16

**Authors:** Kelly Louise Bennett, Martha Kaddumukasa, Fortunate Shija, Rousseau Djouaka, Gerald Misinzo, Julius Lutwama, Yvonne Marie Linton, Catherine Walton

**Affiliations:** ^1^ Faculty of Life Sciences Computational Evolutionary Biology Group University of Manchester Manchester UK; ^2^ Department of Arbovirology, Emerging and Re‐emerging Infections Uganda Virus Research Institute Entebbe Uganda; ^3^ WITS Institute for Malaria Research School of Pathology Faculty of Health Sciences University of Witwatersrand Parktown Johannesburg; ^4^ Department of Veterinary Microbiology and Parasitology Sokoine University of Agriculture Morogoro Tanzania; ^5^ Agro‐Eco‐Health Platform for West and Central Africa International Institute for Tropical Agriculture Cotonou Republic of Benin; ^6^ Department of Entomology National Museum of Natural History Smithsonian Institution Washington DC USA; ^7^ Walter Reed Biosystematics Unit Smithsonian Institution Museum Support Center Suitland MD USA; ^8^ Walter Reed Army Institute of Research Silver Spring MD USA; ^9^ Uniformed Services University of Health Sciences Bethesda MD USA

**Keywords:** *Aedes* mosquitoes, African phylogeography, biodiversity, climate Change, comparative Biology, population genetics–empirical

## Abstract

The study of demographic processes involved in species diversification and evolution ultimately provides explanations for the complex distribution of biodiversity on earth, indicates regions important for the maintenance and generation of biodiversity, and identifies biological units important for conservation or medical consequence. African and forest biota have both received relatively little attention with regard to understanding their diversification, although one possible mechanism is that this has been driven by historical climate change. To investigate this, we implemented a standard population genetics approach along with Approximate Bayesian Computation, using sequence data from two exon‐primed intron‐crossing (EPIC) nuclear loci and mitochondrial cytochrome oxidase subunit I, to investigate the evolutionary history of five medically important and inherently forest dependent mosquito species of the genus *Aedes*. By testing different demographic hypotheses, we show that *Aedes bromeliae* and *Aedes lilii* fit the same model of lineage diversification, admixture, expansion, and recent population structure previously inferred for *Aedes aegypti*. In addition, analyses of population structure show that *Aedes africanus* has undergone lineage diversification and expansion while *Aedes hansfordi* has been impacted by population expansion within Uganda. This congruence in evolutionary history is likely to relate to historical climate‐driven habitat change within Africa during the late Pleistocene and Holocene epoch. We find differences in the population structure of mosquitoes from Tanzania and Uganda compared to Benin and Uganda which could relate to differences in the historical connectivity of forests across the continent. Our findings emphasize the importance of recent climate change in the evolution of African forest biota.

## 1 INTRODUCTION

Understanding the evolutionary processes contributing to the generation and maintenance of biodiversity within Africa's forests is paramount for their future conservation. Although biodiversity has not been characterized for the majority of taxonomic groups, it is clear that the forests of Africa hold a vast amount of species diversity and endemism with important areas including the Guineo‐Congolian rainforest in West and Central Africa, the montane forests of the Albertine rift in Central Africa and the Eastern Arc mountains and coastal forests of Kenya and Tanzania in East Africa (Myers, Mittermeier, Mittermeier, da Fonseca, & Kent, 2000; Scholes et al., 2006). Despite their global importance, the flora and fauna of Africa's forests are largely understudied (Bowie, Fjeldså, Hackett, Bates, & Crowe, 2006; Hewitt, 2004a,b; Plana, 2004) while deforestation and human development currently threaten these habitats (Green et al., 2013; Norris et al., 2010). In addition, forested areas transected by roads lead to sprawling urbanization, bringing human populations into increased contact with forest cycles of disease transmission, including emergent zoonotic arboviruses vectored by mosquitoes (Gubler, 1998; Sang & Dunster, 2001; Vasilakis, Cardosa, Hanley, Holmes, & Weaver, 2011; Walsh, Molyneux, & Birley, 1993; Weaver & Reisen, 2010; Wilcox & Ellis, 2006).

The distribution of biodiversity in Africa is explained to a large extent by the impact of past geological and climatic events (Fjeldså & Bowie, 2008; Hewitt, 1996, 2004a; Plana, 2004). Although Africa has remained relatively geologically stable throughout recent history, with major topographical features in place by the Miocene, the continent has been subject to a turbulent climatic past. In particular, cyclic climate forcing throughout the Plio‐Pleistocene promoted widespread changes in the distribution of habitats. Pollen and sediment cores have shown that tropical forests and wooded grasslands were periodically replaced with savannah during dry glacial phases (deMenocal, 2004; Trauth, Larrasoaña, & Mudelsee, 2009). Fragmentation of the forests at this time may have isolated forest‐dependent species in refugial areas where they were subject to allopatric divergence. When the forests expanded once more with the onset of warm interglacial climate cycles, providing opportunities for range expansions and secondary contact were created. This process of population contraction and expansion, known as the refuge hypothesis (Haffer, 1969), has been used to explain why African forest taxa often have disjunct species distributions separated by unsuitable habitat (Bowie et al., 2006).

Phylogeographic studies have revealed the importance of historical climate change for the diversification of Africa's forest flora and fauna (Hewitt, 2004a,b). Evidence suggests that forest regions have been separated for sufficient time periods for diversification to take place, including divergence events in forest taxa dating to the Plio‐Pleistocene 5 million to 12,000 years ago (Bowie et al., 2006; Hassanin et al., 2015; Huhndorf, Kerbis Peterhans, & Loew, 2007; Mark & Osmaston, 2008; McDonald & Daniels, 2012; Measey & Tolley, 2011; Plana, Gascoigne, Forrest, Harris, & Pennington, 2004; Quérouil, Verheyen, Dillen, & Colyn, 2003; Tosi, 2008) and population structure associated with hypothesized refugial zones in Gabon, Cameroon, and the Lower and Upper Guinea regions of the Guineo‐Congolian rainforest (Anthony et al., 2007; Clifford et al., 2004; Daïnou et al., 2010; Duminil et al., 2010; Koffi, Hardy, Doumenge, Cruaud, & Heuertz, 2011; Lowe, Harris, Dormontt, & Dawson, 2010; Plana et al., 2004). Complementary to this, signals of population expansion (Anthony et al., 2007; Bowie, Fjeldså, Hackett, & Crowe, 2004; Bowie et al., 2006; Jensen‐Seaman & Kidd, 2001; Kebede, Ehrich, Taberlet, Nemomissa, & Brochmann, 2007; Nicolas et al., 2008) and evidence for secondary contact suggest forests were also at times much more expansive including connections between forests in East and West Africa, which are currently separated by dry savannah habitat (Bowie et al., 2004; Couvreur, Chatrou, Sosef, & Richardson, 2008; Kadu et al., 2013; Matthee, Tilbury, & Townsend, 2004; Measey & Tolley, 2011; Wagner, Köhler, Schmitz, & Böhme, 2008).

The forests of Africa support a diverse mosquito fauna that provides a promising system in which to study the effects of climate‐related historical habitat change. This includes members of the genus *Aedes* (Wilkerson & Linton, 2015; Wilkerson et al., 2015) which comprise a number of medically important species. Understanding the diversity of this genus is important to elucidate disease transmission. One such species is *Aedes aegypti* (Linnaeus, 1762), which is a primary vector for several arthropod‐borne viruses (arboviruses) including dengue (DENV), yellow fever (YFV), chikungunya (CHIKV), Venezuelan Equine Encephalitis (VEE), and Zika (ZIKAV) (Germain, Monath, Bryan, Salaun, & Renaudet, 1980; Gubler, 2006; Marchette, Garcia, & Rudnick, 1969; Weaver, 2014). Previous studies providing insight into the large‐scale genetic structure of *Ae. aegypti* have shown that the ancestral African populations are genetically distinct from a more domesticated pantropical form of *Ae. aegypti* that is widespread throughout the Tropics (Bennett et al., 2016; Brown, McBride, et al., 2011, 2014; Crawford et al., 2017; Gloria‐Soria et al., 2016). Inference of the demographic history of *Ae. aegypti* within Africa from nuclear sequence data revealed that this could have been influenced by past climatic change with historical divergence and admixture dated to the late Pleistocene and early Holocene, consistent with demography being shaped by historical changes in forest distribution (Bennett et al., 2016). These findings raise the question of whether past climate change was also important for other African *Aedes*, for which the phylogeographic history is currently unknown.

In addition to the medically important *Ae. aegypti*, three of the four other species investigated here are also arboviral vectors: *Ae. bromeliae* (Theobald 1911) (YFV), *Ae. africanus* (Theobald 1901) (Babanki (BBKV), Bouboui (BOUV), CHIK, Rift Valley Fever (RVFV), YFV, and ZIKAV), and *Aedes hansfordi* (Huang, 1977) (RVFV) (Smithburn, Haddow, & Gillett, 1948; see review in Wilkerson et al., 2015). Sylvatic disease transmission cycles maintained by these *Aedes* species can seed disease epidemics and transfer novel viral genotypes into the human disease transmission cycle (Demanou et al., 2010; Diallo et al., 2005, 2014; Ellis & Barrett, 2008; Mutebi & Barrett, 2002; Ngoagouni et al., 2012; Pastorino et al., 2004; Vasilakis et al., 2011; Wamala et al., 2012). Also included in our study is *Aedes lilii* (Theobald, 1910)*,* which although not currently known to transmit disease to humans, is the sister taxa of *Ae. bromeliae* (*Aedes simpsoni* complex) (Huang, 1979, 1986) yet has a vastly different geographical distribution (Bennett et al., 2015).

Our primary aim is to determine whether historical climate change has impacted the evolutionary history of African *Aedes* mosquitoes. We use a comparative phylogeographic approach to investigate the population histories of four species of *Aedes* (*Stegomyia*) mosquitoes—*Ae. africanus*,* Ae. bromeliae*,* Ae. hansfordi,* and *Ae. lilii*—and compare these to that previously reported for *Ae. aegypti* (Bennett et al., 2016). We take this approach because congruent population histories of across a number of sister taxa provide evidence for a common historical cause (Bermingham & Moritz, 1998). In addition, we use multiple independent molecular markers: two exon‐primed intron‐crossing (EPIC) nuclear loci and mitochondrial *COI* because these different classes of molecular markers resolve different levels of the phylogenetic tree (Huelsenbeck, Bull, & Cunningham, 1996). Demographic signals relating to past climate change can be complex since allopatric divergence may have occurred multiple times in several different refugia, which can be further complicated by periods of secondary contact, founding events, and genetic drift (Estoup & Guillemaud, 2010; Guillemaud, Beaumont, Ciosi, Cornuet, & Estoup, 2009). Therefore in addition to a standard population genetics approach, we use Approximate Bayesian Computation (ABC) to test our hypotheses since this method has been used to successfully link historical climate change to the demographic structure retained in contemporary populations (Barrientos et al., 2014; Fresia, Azeredo‐Espin, & Lyra, 2013; Inoue, Monroe, Elderkin, & Berg, 2014; Logossa et al., 2011; Rovito, 2010).

## 2 METHODS

### 2.1 Mosquito collection and identification

**Figure 1:**
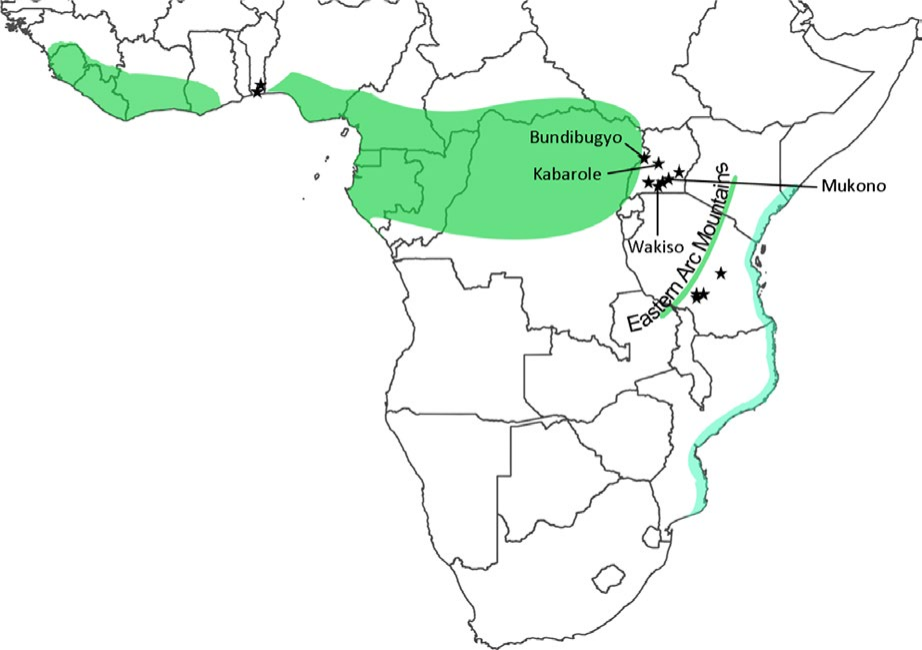
Map of sampling locations within Africa (black stars) in relation to the main Guineo‐Congolian rainforest block (in green) and eastern coastal forests (in blue)

Mosquito larvae were collected from artificial and natural breeding sites including treeholes and plant axils of banana (*Musa* spp.), *Colocasia* spp*.,* and *Dracaena* spp. from Uganda (*n* = 153), Tanzania (*n* = 64), and the Republic of Benin (*n* = 67) (Figure 1 and Table S1). When possible, larvae from each discrete habitat were reared to adulthood as a separate collection because these sites are likely to include siblings; a single individual from each larval collection was selected for genetic analysis to avoid biasing population genetic parameters. Mosquitoes reared to adulthood were pinned or stored in BEEM capsules and desiccated with silica to preserve their DNA. Alternatively, larvae were stored in 100% ethanol. Adult mosquitoes were identified using a morphological key (Huang, 2004) and checked for genetic congruence using phylogenetic tree construction and Bayesian implementation of the general mixed Yule‐coalescent (Reid & Carstens, 2012). As *Ae. bromeliae* and *Ae. lilii* cannot be reliably identified based on morphology (Jupp & Kemp, 1999; Lutwama & Godfrey, 1994), mosquitoes of the Simpsoni Complex were distinguished using a PCR‐identification method based on variation at the internal transcribed spacer region (Bennett et al., 2015).

### 2.2 Experimental procedures

DNA was extracted from larvae or the legs of the adult using a phenol–chloroform extraction method detailed in Surendran et al. (2013). Mosquito DNA was sequenced at two nuclear loci using the exon‐primed intron‐crossing (EPIC) markers *IDH2* (with primers 5′‐CAGCAGTGCGTTCTTTTTCC‐′3 and 5′‐CAGTATCGATGCCCTTGTGG‐′3) developed in Bennett et al. (2016), and *RpL30b* described in White, Endersby, Chan, Hoffmann, and Weeks (2014). PCR products for direct sequencing were generated with 1.25 units of BIOTAQ™ DNA Polymerase (BioLine, UK), 1× NH_4_ Reaction Buffer, 2 mM MgCl_2_, 0.8 mM of each dNTP, 0.5 μM forward and reverse primer, and 1–10 ng of template DNA in a total volume of 25 μl. The thermocycling conditions were 95°C for 2 min followed by 35 cycles of 95°C for 15 s, 60°C for 30 s, 72°C for 30 s, and a final elongation step at 72°C for 10 min. Folmer, Black, Hoeh, Lutz, and Vrijenhoek's (1994) universal primers LCO1490 (5′‐GGTCAACAAATCATAAAGATATTGG‐′3) and HCO2198 (5′‐TAAACTTCAGGGTGACCAAAAAATCA‐′3) were used to amplify a portion of the mitochondrial gene *COI* as detailed by the Consortium for the Barcode of Life (http://barcoding.si.edu/dnabarcoding.htm). The GenElute PCR Clean‐Up Kit (Sigma‐Aldrich Co. LLC, UK) was used to purify PCR products. The BigDye Terminator v3.1 cycle sequencing kit was used to sequence the targeted regions in both forward and reverse directions on a 3,730 automated Sanger sequencer (Applied Biosystems, UK).

PCR products were cloned when indels between homologous alleles within an individual obscured the sequence read in the direct sequence of a PCR product and when sequences had low haplotype phase probability. PCR products for cloning were generated with high fidelity MyFi polymerase (BioLine, UK), 1× MyFi Reaction Buffer, 0.8 μM forward and reverse primer, and 1–10 ng of template DNA. To reduce the formation of chimeras (Smyth et al., 2010; Stevens, Jackson, & Olson, 2013), a 30% ramp speed was used to implement the following thermal cycle; 95°C for 3 min followed by 30 cycles of 95°C for 30 s, 62°C for 45 s, and 72°C for 45 s with no final extension. PCR products were purified as above and cloned using the pGEM‐T vector system (Promega, UK) according to kit instructions. Product inserts were amplified with universal M13 primers (5′‐TGTAAAACGACGGCCAGT‐′3 and 5′‐CAGGAAACAGCTATGAC‐′3) (Messing, 1983) using the BIOTAQ™ DNA Polymerase and sequencing protocol described above.

### 2.3 Data analysis

The mitochondrial sequences of *Ae. aegypti* included in the Africa‐wide analyses were those analyzed in Bennett et al. (2016) while sequences included in regional analyses are detailed in Table S1. The sequences of nuclear genes of *Ae. aegypti* were those generated previously (GenBank references KX444686–KX446391 in Bennett et al., 2016). Mitochondrial sequences for members of the Simpsoni Complex generated during this study were analyzed together with those previously reported in Bennett et al., 2015 (GenBank KT998389–429). For all sequence data, compound indels affecting less than 5% of the sequence alignment were removed to eliminate areas with uncertain sequence homology in Geneious v5.4.7 (Kearse et al., 2012). A *COI* sequence of one individual of the Simpsoni Complex was removed from analysis because double peaks were present in its ABI trace files which signifies coamplification of nuclear mitochondrial DNA (NUMT's) (Bensasson, Zhang, Hartl, & Hewitt, 2001; Hlaing et al., 2009). To check for indications of NUMT's, the remaining *COI* sequences were translated to detect any stop codons and to ensure that the Ka/Ks ratio was as expected in MEGA6 (Tamura, Stecher, Peterson, Filipski, & Kumar, 2013). Nuclear haplotypes were reconstructed with PHASE v2.1.1 and accepted if above a probability threshold of 0.8. Otherwise, haplotypes were resolved through cloning as described above or removed from analysis if comprising less than 2% of the total dataset as recommended by Garrick, Sunnucks, and Dyer (2010).

### 2.4 Tests of recombination

Tests to detect recombination included the pairwise homoplasy test (φ) and Neighbour Similarity Score implemented in PhiPack (Bruen, Philippe, & Bryant, 2006) and Max chi‐square, Chimaera, and GENECONV performed in RDP4 (Martin et al., 2010; Padidam, Sawyer, & Fauquet, 1999; Posada & Crandall, 2001; Smith, 1992).

### 2.5 Phylogenetic trees

Phylogenetic trees for each genetic locus were calculated in MEGA7 (Kumar et al. 2016) using the best available substitution models as chosen by jModelTest (Figures S1, S2, S3 (Posada, 2008).

### 2.6 Analyses of diversity and population structure

DNAsp v5 (Librado & Rozas, 2009) was used to generate nucleotide diversity per site (π), Watterson's theta (θ_w_), and the number of segregating sites (S). Statistics to detect demographic expansion, Tajima's *D* and Fu's Fs, and genetic differentiation, *F*_ST_, were generated in Arlequin v3.5 (Excoffier & Lischer, 2010). A Mantel test for correlation between genetic and geographical distance was used to test for isolation by distance (IBD) in R v3.1.3 (R Core Team 2015) using the packages Adegenet (Jombart, 2008) and APE (Paradis, Claude, & Strimmer, 2004). Effective population sizes were calculated from the joint estimation of Ɵ for nuclear markers *IDH2* and *RpL30b* assuming Ɵ = 4N_e_μ, using the Bayesian implementation of LAMARC. For μ, we used the nuclear substitution rate of 1.6 × 10^−9^ substitutions per site per generation (Moriyama & Gojobori, 1992) and 5.8 × 10^−9^ mutations per site per generation (Haag‐Liautard et al., 2007) for *Drosophila* assuming a generation time of 0.1 years (Cutter, 2008; Irvin, Hoddle, O'Brochta, Carey, & Atkinson, 2004; Sowilem, Kamal, & Khater, 2013). Two independent runs of three replicates were run with an initial burn‐in of 100,000 steps followed by 10,000,000 iterations.

### 2.7 Haplotype networks

Median‐joining networks were constructed for sequence datasets in NETWORK v4.6.1.3 (Fluxus Technology Ltd.). Reticulation within networks was resolved using the recommendations in Crandall and Templeton (1993). Haplotypes with missing data were removed for construction of mitochondrial *COI* networks for *Ae. lilii* (*n* = 1), *Ae. africanus* (*n* = 1), and *Ae. hansfordi* (*n* = 1) because their inclusion increased network complexity (Joly, Stevens, & van Vuuren, 2007). Minimum and maximum pairwise differences within each genetic locus and mitochondrial *COI* haplogroup were calculated in MEGA7 (Kumar et al. 2016) using a Tamura and Nei substitution model with uniform rates across sites (Table S5).

### 2.8 Bayesian GMRF Skyride analysis

Datasets were tested for population size expansion using the GMRF (Gaussian Markov random field) skyride model (Minin, Bloomquist, & Suchard, 2008) in BEAST v1.8.1 (Drummond, Suchard, Xie, & Rambaut, 2012). This model is an extension of the standard Bayesian skyline function that estimates effective population size for a series of intervals based on the number of coalescent events while incorporating a smoothing function to penalize population size changes. In addition, a user defined prior on the number of population size changes is not required. To avoid violating the assumption of panmixia assumed by GMRF skyride, individuals from divergent *COI* clades within *Ae. bromeliae*,* Ae. lilii*,* Ae. africanus,* and *Ae. aegypti* were used in separate analyses. GMRF skyride plots were generated with 1,000,000,000 Markov chains sampled every 100,000 generations. Each sequence set was tested for conformity to a molecular clock model in MEGA6 using a Likelihood Ratio Test (Tamura et al., 2013), and all were found to reject the null hypothesis of a constant evolutionary rate among branches. Therefore, a lognormal relaxed clock was used to generate trees with substitution rate priors. For nuclear loci, the lower estimate was placed at 1.6 × 10^−9^ substitutions per site per generation (Moriyama & Gojobori, 1992) and the upper estimate at 5.8 × 10^−9^ per site per generation (Haag‐Liautard et al., 2007) estimated for *Drosophila* assuming a generation time of 0.1 years (Cutter, 2008; Irvin et al., 2004; Sowilem et al., 2013). For mitochondrial *COI,* a lower prior of 1.15 × 10^−8^ (Brower, 1994) and an upper prior of 1.27 × 10^−8^ substitutions per site per generation (Papadopoulou, Anastasiou, & Vogler, 2010) were used. Outputs were visualized in LogCombiner v1.5 (Rambaut, Suchard, Xie, & Drummond, 2009) to assess convergence before generating GMRF Skyride plots using the inbuilt application.

### 2.9 Approximate Bayesian computation

**Figure 2:**
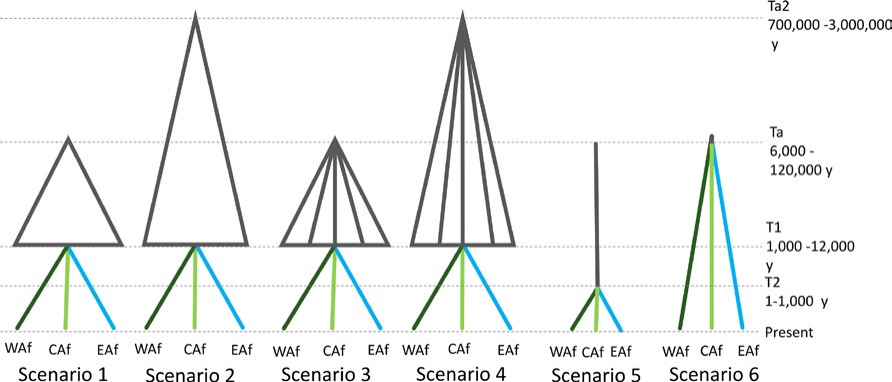
Six competing evolutionary scenarios for three populations, Benin, West Africa (WAf), Uganda, Central Africa (CAf) and Tanzania, East Africa (Neafsey et al., 2015), tested within DIYABC

Consistent with our comparative phylogeographic approach, we apply here the same six evolutionary scenarios used in Bennett et al. (2016) to test alternative hypotheses of the impact of past climate‐driven habitat change in Africa on the demographic history of *Aedes aegypti* (Figure 2). These scenarios were based on available genetic data *for Ae. aegypti* as well as information on African forest refugia. Scenarios 1–4 involve divergence in forest refugia followed by admixture during interglacials. Scenario 1, in which populations diverged in allopatry in two major forest refugia during the most recent glacial periods of the late Pleistocene, is based on the presence of two distinctive mitochondrial haplogroups in African *Ae. aegypti* (Moore et al., 2013). Recent glacial periods occurred in between interglacial periods estimated at 5–11,000, 45–50,000, and 110–120,000 years ago (Castañeda et al., 2009). Therefore, priors for allopatric divergence were set at 6,000–120,000 years (Ta) ago to encompass this range. Scenario 1 also considers that after allopatric divergence, populations were subject to admixture during the last of these interglacial periods (T1) (Castañeda et al., 2009). Since interglacials occurred periodically, mosquitoes could have undergone multiple admixture events throughout the late Pleistocene. Related divergence could have led to the speciation of sister taxa *Ae. lilii* and *Ae. bromeliae*, relatively recently as indicated by incomplete lineage sorting in the phylogenetic trees (Figures S1, S2 and S3). However, because admixture is likely to complicate the inference of earlier events (Bennett et al., 2016 and references herein), we only consider the most recent time periods and therefore analyze *Ae. lilii* and *Ae. bromeliae* separately rather than as a single taxon.

Based on estimates of mitochondrial divergence in *Ae. aegypti*, allopatric divergence into habitat refugia could have also occurred earlier, during the mid‐Pleistocene, 700,000 to 3 million years ago (Ta2) (Bennett et al., 2016). Therefore, we include scenario 2 to consider deep allopatric divergence into two forest refugia during this time. As in scenario 1, scenario 2 additionally models admixture during the last interglacial period. Scenarios 3 and 4 were designed to account for allopatric divergence into many multiple refugia during either the mid‐ (Ta2) or late Pleistocene (Ta), respectively. Numerous refugia are postulated to have persisted in West and Central Africa (Diamond & Hamilton, 1980; Maley, 1991; Ray & Adams, 2001) and in the East African Rift mountains of Kenya and Eastern Arc mountains of Kenya and Tanzania (Diamond & Hamilton, 1980; Fjeldså, Johansson, Lokugalappatti, & Bowie, 2007; Maley, 1991). Therefore, both scenarios 3 and 4 model allopatric divergence into five independent populations, to capture a reasonable level of this potential divergence, before admixture during the last interglacial period. In addition to their specific features, all the afore‐mentioned models (scenarios 1–4) include a period of recent population divergence occurring during the last 1,000 years to account for geographic structure and to allow the inclusion of regional groups. These four models were tested against alternative demographic scenarios hypothesized for mosquito populations which were not impacted by past climate change. Scenario 5 considers that populations of *Aedes* have remained panmictic through history without past events of divergence or admixture. Recent divergence within the last 1,000 years is included in this model to allow comparison with other models in the same analysis. Finally, scenario 6 models independent divergence of populations without admixture occurring during the Holocene climate oscillations.

Tanzanian populations represent East Africa, Ugandan populations represent Central Africa, and the Republic of Benin as West Africa. Maximum effective population sizes of African demes were set to cover a wide range (10,000–14,000,000 individuals) encompassing values based on estimates of Ɵ calculated in DNAsp v5 and LAMARC as described above. For both *Ae. bromeliae* and *Ae. lilii* datasets, the simulation program DIYABC v2.0.4 (Cornuet, Ravigne, & Estoup, 2010) was used to analyze both nuclear loci together in the same analysis and to analyze mitochondrial *COI* sequences separately. In addition, the sequence data of each nuclear loci were also analyzed separately to test for concordance with the result of the joint ABC analysis of both nuclear loci. ABC analysis was not performed for *Ae. aegypti* in this study, since the above demographic scenarios have been tested for the same molecular loci previously (Bennett et al., 2016). In addition, this analysis was not applied to the *Ae. africanus* and *Ae. hansfordi* datasets because sample sizes were low. Indeed, test runs for each species dataset suggested that model inference was inconclusive, with multiple models achieving a high posterior probability. For each evolutionary scenario tested for *Ae. lilli* and *Ae. bromeliae*, 6,000,000 simulations were performed. A range of priors implemented in preliminary runs was used to assess the ability of simulations to produce statistics close to observed values. Prior ranges are given in Table S2. Logistic regression (Cornuet et al., 2010) was used to compare the closest 1% of simulated values to observed data and to estimate posterior probabilities of scenarios. Summary statistics chosen to compare scenarios included the number of segregating sites (S) and mean and variance of pairwise differences as one sample summary statistics within populations. Two sample summary statistics included the mean of pairwise differences and *F*_ST_ between populations. Model checking was used to evaluate confidence in the posterior probabilities of scenarios. To implement this, 1,000 pseudo‐observed datasets were generated from the posterior distribution of parameters. When possible, model fit was assessed with different statistics to those used for model inference as recommended by Cornuet et al. (2010). These statistics included Tajima's *D* and private segregating sites within populations and the number of segregating sites and mean of pairwise differences (B) between populations. Posterior based error rates were calculated using the scenario choice and parameter values of the closest 500 simulated datasets to the observed data.

## 3 RESULTS

### 3.1 Tests of recombination

No recombination was detected for the nuclear sequence datasets with the tests implemented in RDP4. However, significant homoplasy compatibility scores (φ) were detected at nuclear *IDH2* for *Ae. bromeliae* (*p *=* *.001), *Ae. hansfordi* (*p *=* *.02), and *Ae. africanus* (*p *=* *.03) and at nuclear *Rp30Lb* for *Ae. africanus* (*p *=* *.01). These datasets were trimmed by approximately 20–50 bp to remove signals of recombination for phylogenetic analyses, giving a reduced sequence length of 357 bp at *IDH2* for *Ae. bromeliae* with a nonsignificant homoplasy score (*p *=* *.28), 501 bp at *IDH2* for *Ae. hansfordi* (*p *=* *.37), 426 bp at *IDH2* for *Ae. africanus* (*p *=* *.21), and 252 bp at *Rp30Lb* for *Ae. africanus* (*p *=* *.26).

### 3.2 Analyses of diversity and population structure

All five *Aedes* species have high levels of genetic diversity (Ɵ and π) and effective population sizes within Africa that are generally comparable across species (Tables 1 and 2). Reduced genetic diversity at the *IDH2* locus in *Ae. aegypti* compared with other species and markers could indicate that this gene is under positive selection. This was tested previously with the HKA test and narrowly failed to be significant (Bennett et al., 2016). A HKA test comparing the lower genetic diversity at the *IDH2* locus in *Ae. lilii* to that of *Ae. hansfordi* and nuclear *RpL30b* was nonsignificant (χ^2^ = 1.10, *p *=* *.30). Genetic diversity (Ɵ and π) was lower in Benin than Tanzania and Uganda for Ae. *lilii* and *Ae. aegypti* (the only two species sampled in Benin) for all loci except *COI* of *Ae. lilii*. Tajima's *D* was significant and negative within Africa at one or more nuclear loci for all species except *Ae. africanus* (Table 1). Significant negative Tajima's *D* values were at *RpL30b* for all the Africa‐wide analyses of *Ae. bromeliae*,* Ae. lilii,* and *Ae. aegypti* and for all but one regional population grouping of these species (*Ae. aegypti*, Benin). By comparison, the *IDH2* locus had fewer significantly negative Tajima's *D* values, but they were present for the Africa‐wide analyses of *Ae. lilii* and *Ae. aegypti* as well as for Ugandan *Ae. aegypti, Ae. bromeliae,* and *Ae. hansfordi*. With the exception of *Ae. lilii* in Uganda, all Tajima's *D* values for mitochondrial *COI* were nonsignificant. Similarly, significant Fu's Fs values were seen in Africa and regional populations for all species including *Ae. africanus* (Table 1). Significant Fu's Fs were also seen at the mitochondrial locus in Ugandan *Ae. hansfordi* and Africa‐wide *Ae. aegypti* (Table 1).

**Table 1:** Table of summary statistics for each species and genetic loci including number of sequences (No. seqs), number of haplotypes (No. haps), number of segregating sites (S), Watterson's theta (Ɵw), and nucleotide diversity (π)

Species	Gene	Region	No. seqs	No.haps	S	Ɵw	π	Tajima's *D*	P value	Fu's Fs	P value
*Aedes bromeliae*	*IDH2*	Africa	82	47	45	0.0253	0.0143	−1.40	0.06	−**25.48**	**0.00**
		Tanzania	52	31	26	0.0174	0.0139	−0.44	0.36	−**19.97**	**0.00**
		Uganda	30	18	33	0.0233	0.0135	−**1.53**	**0.03**	−**7.02**	**0.00**
	*RpL30b*	Africa	90	38	37	0.0290	0.0080	−**2.02**	**0.00**	−**26.07**	**0.00**
		Tanzania	60	26	28	0.0236	0.0075	−**1.87**	**0.01**	−**14.56**	**0.00**
		Uganda	30	22	23	0.0220	0.0130	−**1.66**	**0.02**	−**6.43**	**0.01**
	*COI*	Africa	53	15	18	0.0085	0.0123	1.39	0.93	−0.43	0.50
		Tanzania	33	8	7	0.0037	0.0000	0.68	0.78	−0.90	0.34
	* *	Uganda	20	7	11	0.0066	0.0048	−1.00	0.16	−0.88	0.33
*Aedes lilii*	*IDH2*	Africa	86	30	27	0.0170	0.0080	−**1.63**	**0.03**	−**16.50**	**0.00**
		Benin	44	17	18	0.0116	0.0087	−0.81	0.25	−**6.59**	**0.00**
		Uganda	42	13	19	0.0124	0.0071	−1.40	0.06	−**3.90**	**0.04**
	*RpL30b*	Africa	86	36	37	0.0290	0.0083	−**2.26**	**0.00**	−**26.98**	**0.00**
		Benin	42	18	20	0.0181	0.0063	−**2.09**	**0.00**	−**14.75**	**0.00**
		Uganda	44	27	29	0.0265	0.0109	−**1.99**	**0.00**	−**20.17**	**0.00**
	*COI*	Africa	53	8	19	0.0094	0.0104	0.42	0.72	3.74	0.91
		Benin	24	5	16	0.0096	0.0142	1.82	0.97	6.60	0.99
	* *	Uganda	29	5	17	0.0093	0.0044	−**1.83**	**0.02**	1.32	0.78
*Aedes africanus*	*IDH2*	Uganda	60	28	40	0.0212	0.0133	−1.06	0.14	−**9.06**	**0.01**
	*RpL30b*	Uganda	76	37	28	0.0227	0.0122	−1.19	0.12	−**25.96**	**0.00**
	*COI*	Uganda	49	6	21	0.0157	0.0244	1.81	0.98	9.37	0.99
*Aedes hansfordi*	*IDH2*	Uganda	26	25	42	0.0225	0.0123	−**1.67**	**0.03**	−**21.93**	**0.00**
	*RpL30b*	Uganda	28	22	23	0.0217	0.0131	−1.43	0.07	−**18.00**	**0.00**
	*COI*	Uganda	19	6	6	0.0027	0.0015	−1.41	0.07	−**2.44**	**0.02**
*Aedes aegypti*	*IDH2*	Africa	270	51	49	0.0190	0.0073	−**1.71**	**0.01**	−**25.80**	**0.00**
		Benin	70	20	23	0.0109	0.0075	−0.90	0.21	−**5.94**	**0.02**
		Tanzania	66	19	25	0.0124	0.0082	−1.01	0.16	−4.32	0.07
		Uganda	56	25	32	0.0157	0.0076	−**1.77**	**0.02**	−**12.94**	**0.00**
	*RpL30b*	Africa	330	74	67	0.0448	0.0104	−**2.13**	**0.00**	−**26.00**	**0.00**
		Benin	86	25	25	0.0187	0.0089	−1.41	0.07	−**8.33**	**0.01**
		Tanzania	60	24	35	0.0278	0.0099	−**2.00**	**0.01**	−**12.21**	**0.00**
		Uganda	82	45	37	0.0298	0.0120	−**1.73**	**0.01**	−**26.46**	**0.00**
	*COI*	Africa	169	29	22	0.0140	0.0066	−0.75	0.28	−**6.62**	**0.05**
		Benin	26	6	6	0.0035	0.0035	−0.52	0.32	1.78	0.84
		Tanzania	22	14	28	0.0156	0.0114	−1.25	0.11	−3.01	0.09
	* *	Uganda	39	14	13	0.0102	0.0068	−0.54	0.35	−1.82	0.24

Significant values of Tajima's *D* and Fu's Fs that deviate from the null hypothesis of neutral mutation (*p* < .05) are shown in bold.

**Table 2:** Theta (θ) estimated over nuclear genes *IDH2* and *RpL30b*, 95% confidence intervals (95% CI) and effective population size estimates (Ne) using two different substitution rates (μ) for species demographic groups

Species	Population	Overall θ	95% CI	Ne (μ* *=* *1.6E‐09)	Ne (μ* *=* *5.8E‐09)
*Aedes hansfordi*	Uganda	0.055	0.041–0.073	8625000	2379311
*Aedes africanus*	Uganda	0.055	0.041–0.146	8609375	2375000
*Aedes bromeliae*	Tanzania	0.053	0.038–0.072	8210938	2265087
	Uganda	0.050	0.033–0.081	7890625	2171225
	Africa	0.094	0.074–0.120	14726563	4062500
*Aedes lilii*	Benin	0.023	0.015–0.035	3625000	1000000
	Uganda	0.043	0.028–0.069	6765625	1866379
	Africa	0.055	0.040–0.071	8601563	2372845
*Aedes aegypti*	Benin	0.024	0.018–0.034	3796875	1047414
	Uganda	0.055	0.038–0.081	8523438	2066811
	Tanzania	0.052	0.045–0.068	4664063	1286638
	Africa	0.083	0.070–0.090	12898438	3558190

On pairwise comparison of all regional populations for all loci within species, there was significant genetic differentiation between populations of *Ae. bromeliae* from Tanzania and Uganda at nuclear loci *IDH2* (*F*_ST_ = 0.074, *p *=* *.00) and *Rp30Lb* (*F*_ST_ = 0.039, *p *=* *.03) and mitochondrial *COI* (*F*_ST_ = 0.778, *p *=* *.00), somewhat higher than the values reported for *Ae. aegypti* previously (*IDH2*,* F*_ST_ = 0.021; *Rp30Lb*,* F*_ST_ = 0.014; *COI*,* F*_ST_ = 0.083). Significant genetic differences were also apparent between populations of *Ae. lilii* in Benin, West Africa, and Uganda, Central Africa at the *COI* gene (*F*_ST_ = 0.269, *p *=* *.00) but not at nuclear loci. Similarly, structure between West and Central Africa was reported previously at *Rp30Lb* (*F*_ST_ = 0.013) and *COI* (*F*_ST_ = 0.075) for *Ae. aegypti*, in addition to structure between East and West African *Ae. aegypti* at nuclear *IDH2* (*F*_ST_ = 0.015), *Rp30Lb* (*F*_ST_ = 0.036), and mitochondrial *COI* (*F*_ST_ = 0.252) (Bennett et al., 2016).

Significant isolation by distance (IBD) was detected at the *COI* locus (*r* = .19, *p *=* *.01) and nuclear *IDH2* (*r* = .09, *p *=* *.01) and *Rp30Lb* (*r* = .11, *p *=* *.01) for *Ae. bromeliae*. In addition, significant IBD was detected at mitochondrial *COI* for *Ae. lilii* (*r* = .20, *p *=* *.01) and *Ae. africanus* (*r* = .13, *p *=* *.02). All other tests for IBD were nonsignificant, as reported for these loci for *Ae. aegypti* previously (Bennett et al., 2016).

### 3.3 Haplotype networks

**Figure 3:**
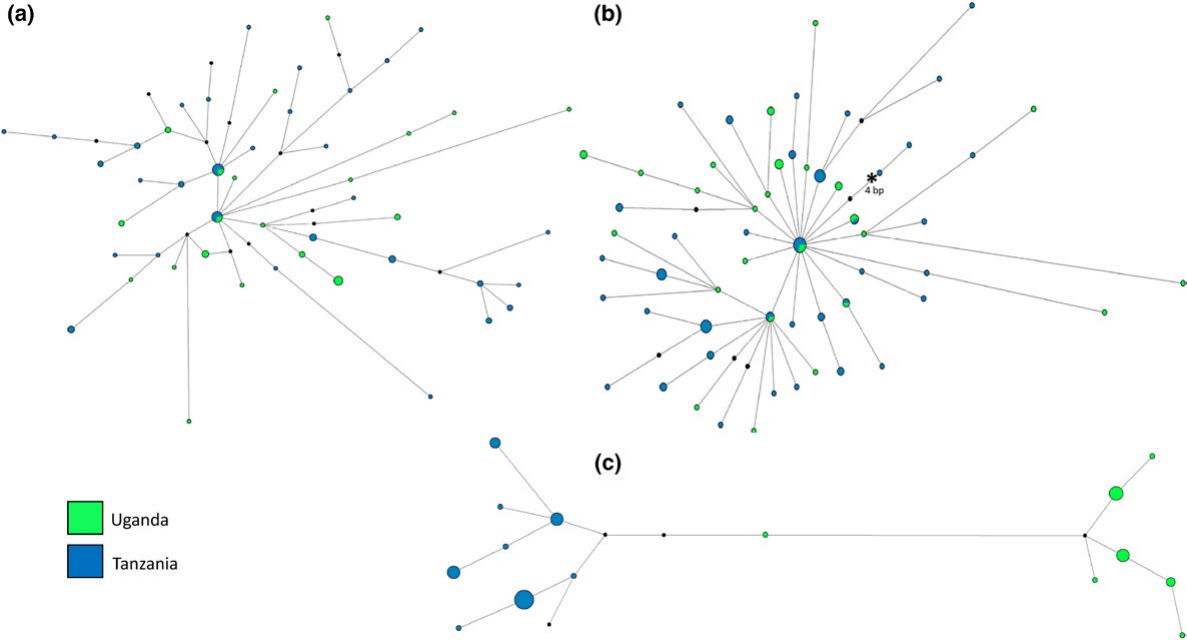
Haplotypes networks for *Aedes bromeliae* nuclear genes (a) *IDH2*, (b) *Rp30Lb,* and mitochondrial (c) *COI*

**Figure 4:**
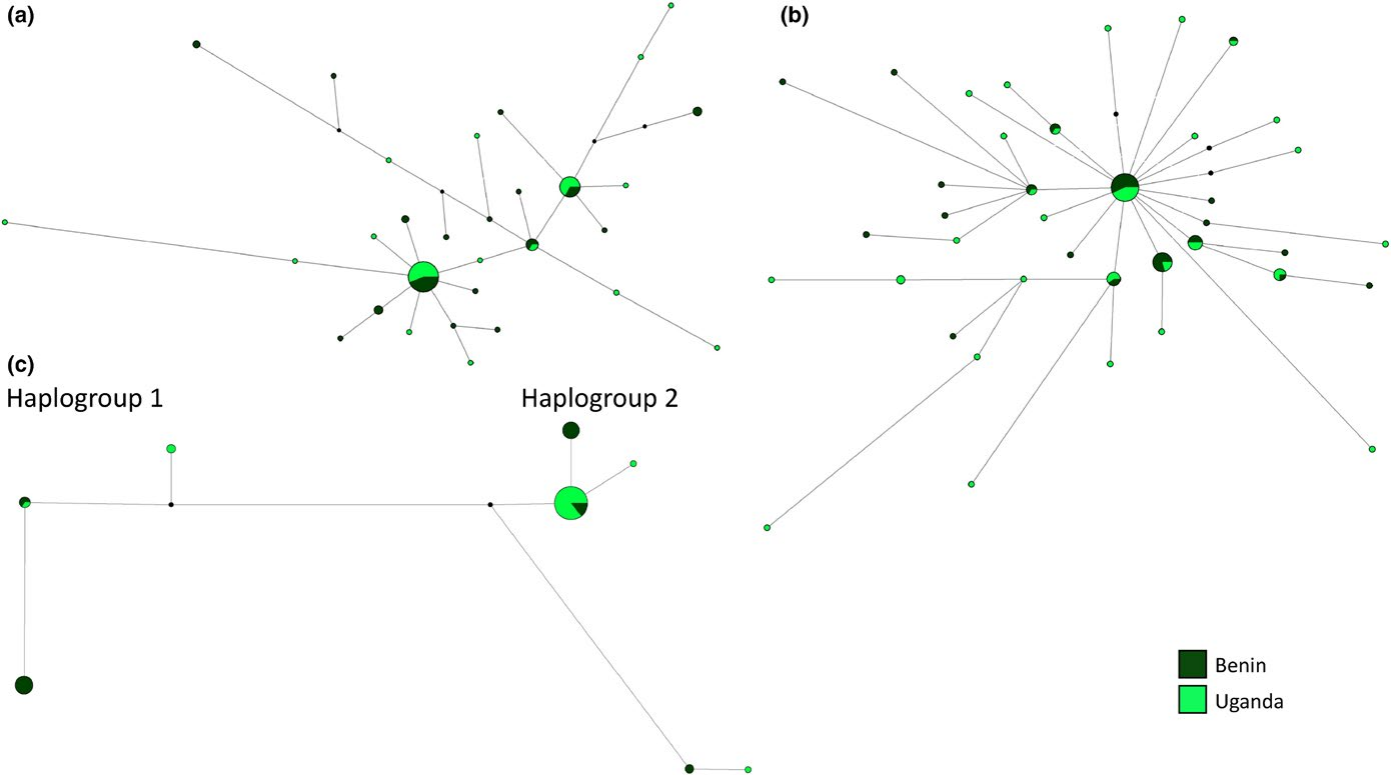
Haplotypes networks for *Aedes lilii* nuclear genes (a) *IDH2*, (b) *Rp30Lb* and mitochondrial, (c) *COI*

The median‐joining network for mitochondrial *COI* from *Ae. bromeliae* revealed a strong geographic structure with two major haplogroups representing Uganda, Central Africa, and Tanzania, East Africa (Figure 3) separated by 10 or 11 mutational steps (uncertainty in number of steps being due to the placement of a median vector before the Ugandan haplotype cluster). The sole exception to this was one Ugandan haplotype from one individual that was only two to three mutational steps away from the Tanzanian cluster. In contrast, the sequence data for the nuclear loci, *IDH2,* and *Rp30Lb* formed a single star‐like cluster of haplotypes in *Ae. bromeliae* (Figure 3). In this network, there was support for some local geographical structuring because haplogroups specific to Uganda or Tanzania radiated from more common haplotypes, which were shared between geographic regions. The haplotype network for *Ae. lilii* and mitochondrial *COI* also revealed two major haplogroups, separated by six mutational steps, each haplogroup representing a mix of haplotypes from Uganda, Central Africa and Benin, West Africa (Figure 4). These two groups were not apparent in the haplotype networks for the nuclear *IDH2* and *Rp30Lb* loci which each form a single haplogroup (Figure 4).

**Figure 5:**
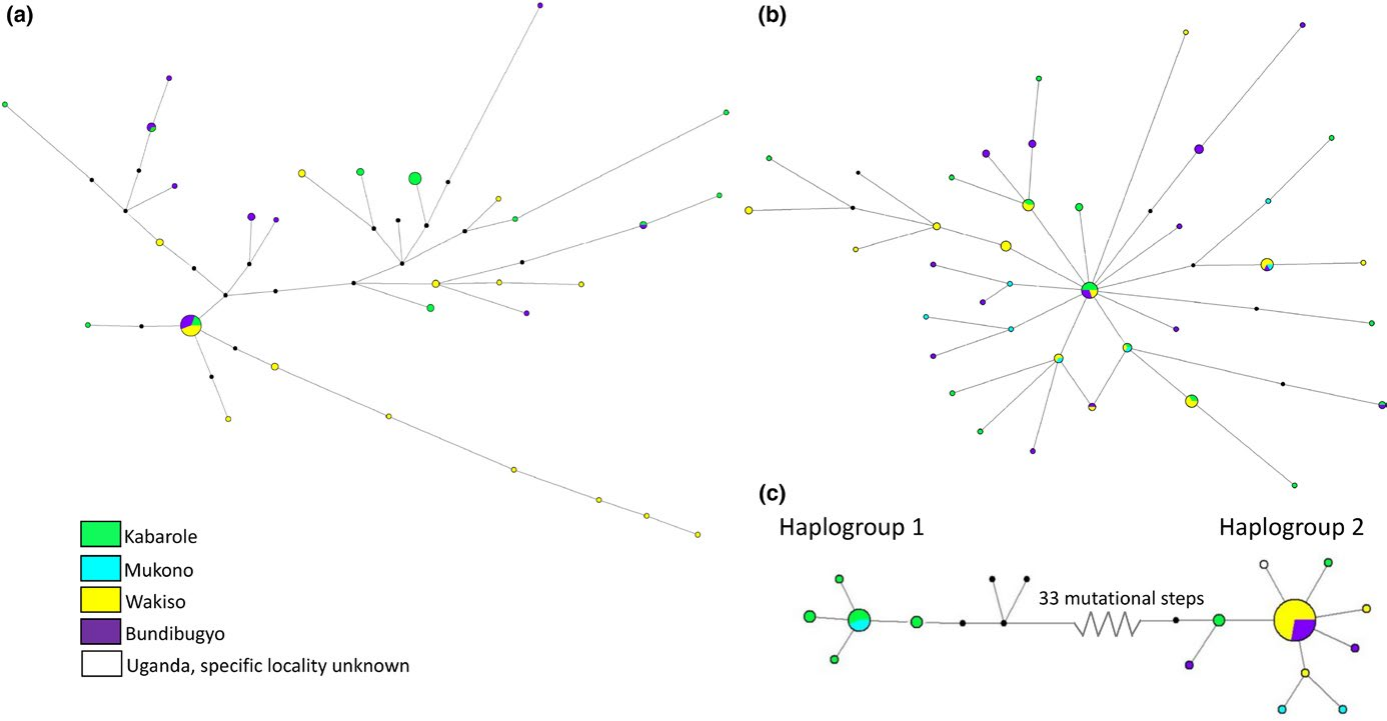
Haplotypes networks for *Aedes africanus* nuclear genes (a) *IDH2*, (b) *Rp30Lb,* and mitochondrial (c) *COI*

The haplotype network of mitochondrial *COI* revealed two highly divergent haplogroups in *Ae. africanus*, separated by 33 mutational steps or 5.8% sequence divergence (Figure 5). These clades broadly represented different Ugandan populations; while haplogroup 1 included individuals only from Kabarole and Mukono, haplogroup 2 included mainly individuals from Bundibugyo and Wakiso, although low numbers of haplotypes from Kabarole and Mukono were also present. In comparison with the mtDNA, there was no major separation of the genetic diversity within the haplotype networks of nuclear *IDH2* or *Rp30Lb* (Figure 5).

**Figure 6:**
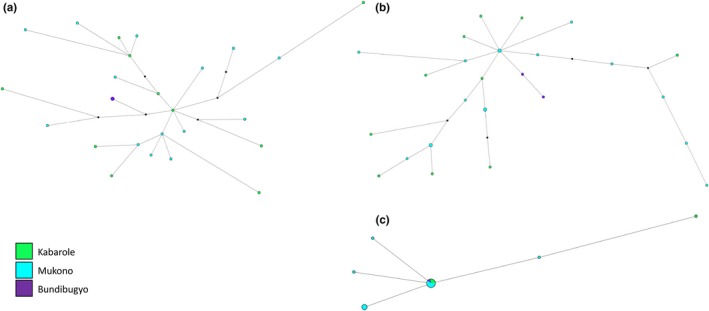
Haplotypes networks for *Aedes hansfordi* nuclear genes (a) *IDH2*, (b) *Rp30Lb,* and mitochondrial (c) *COI*

*Aedes hansfordi*, represented here only by populations from Uganda, comprised a single haplogroup at the *COI* locus (Figure 6). Although haplotype networks for nuclear *IDH2* and *Rp30Lb* revealed a greater genetic diversity compared to mitochondrial data, there was no obvious structuring into haplogroups was evident at these loci (Figure 6).

### 3.4 Bayesian GMRF Skyride analysis

For all species, Bayesian GMRF skyride analysis did not detect a change in population size on analysis of nuclear loci (*IDH2* and *RpL30b*) and mitochondrial *COI* data in the majority of cases. However, a signal of demographic expansion was detected on analysis of *COI* haplogroup 2 for *Ae. lilii* and for *Ae. africanus* with an increase in effective population size from approximately 40,000–2,000,000 from 100,000 years ago and an increase in effective population size from 30,000 to 1,000,000 from 70,000 years ago, respectively.

### 3.5 Approximate Bayesian computation

Approximate Bayesian computation (ABC) analysis of nuclear sequence data, that is, *IDH2* and *RpL30b* together, revealed that scenario 3 was the most probable for *Ae. bromeliae* and *Ae. lilii* on comparison of the posterior probabilities of hypothesized models (Table 3). This chosen scenario models late Pleistocene divergence into five populations occurring between 6,000 and 120,000 years ago, followed by admixture in the Holocene 1,000 to 12,000 years ago and recent population divergence within the last 1,000 years (Figure 2). Individual analysis of nuclear loci agreed with this scenario choice. ABC scenario choice for mitochondrial *COI* identified the highest probability model as scenario 1 for *Ae. lilii*. This model is similar to scenario 3 and only differs in that it simulates allopatric divergence into two ancestral populations rather than five populations. On analysis of mitochondrial *COI* for *Ae. bromeliae,* the highest probability model was scenario 6, which models panmixis followed by recent independent divergence of regional populations. Posterior error rates were fairly high for all analyses (Table 3), possibly because the models tested (scenarios 1 and 3) include common demographic events, that is, scenarios 1 and 3 both include late Pleistocene divergence and early Holocene admixture events. Alternatively, the true population history is more complex than tested, which could be resolved with analysis of more molecular loci and increased sampling. Estimates for demographic and mutational parameters for the selected models are provided in Tables S3 and S4.

**Table 3:** Posterior probabilities (Post.prob) for six hypothesised evolutionary scenarios tested in DIYABC for each species based on joint analysis of nuclear DNA (nDNA), each nuclear loci (*RpL30b*,* IDH2*) and mitochondrial *COI* (mtDNA)

Species	Scenario	nDNA			RpL30b			IDH2			mtDNA		
		Post.prob.	95% CI	Posterior error	Post.prob.	95% CI	Posterior error	Post.prob.	95% CI	Posterior error	Post.prob.	95% CI	Posterior error
*Aedes bromeliae*	Sc1	0.263	0.255–0.270	0.536	0.270	0.262–0.278	0.522	0.234	0.228–0.239	0.592	0.171	0.165–0.176	0.596
	Sc2	0.002	0.000–0.016		0.002	0.000–0.017		0.006	0.000–0.012		0.131	0.126–0.136	
	Sc3	**0.432**	**0.423–0.441**		**0.454**	**0.445–0.463**		**0.358**	**0.352–0.365**		0.153	0.148–0.158	
	Sc4	0.017	0.003–0.031		0.017	0.002–0.032		0.024	0.018–0.030		0.094	0.090–0.099	
	Sc5	0.086	0.066–0.106		0.084	0.063–0.105		0.269	0.261–0.277		0.001	0.000–0.003	
	Sc6	0.200	0.191–0.210		0.174	0.164–0.183		0.110	0.104–0.115		**0.450**	**0.442–0.458**	
*Aede lilii*	Sc1	0.179	0.000–0.000	0.304	0.239	0.231–0.247	0.458	0.238	0.229–0.246	0.422	**0.377**	**0.371–0.382**	0.574
	Sc2	0.000	0.000–0.000		0.002	0.000–0.017		0.004	0.000–0.019		0.062	0.059–0.066	
	Sc3	**0.463**	**0.450–0.476**		**0.512**	**0.504–0.523**		**0.511**	**0.501–0.520**		0.278	0.273–0.283	
	Sc4	0.012	0.000–0.025		0.024	0.010–0.039		0.023	0.009–0.038		0.062	0.059–0.065	
	Sc5	0.230	0.280–0.316		0.100	0.079–0.121		0.104	0.083–0.125		0.150	0.145–0.154	
	Sc6	0.049	0.036–0.062		0.121	0.109–0.133		0.121	0.110–0.133		0.071	0.067–0.076	

95% confidence intervals (95% CI) of posterior probabilities and posterior error rates are given for each analysis. The values for scenarios with the highest posterior probabilities are shown in bold.

## 4 DISCUSSION

Previously, populations of African *Ae. aegypti* were found to fit a pattern of historical lineage diversification and admixture followed by recent population structuring (Bennett et al., 2016). Two explanations were put forward for this population history, the first being the refuge hypothesis of climate‐induced vicariance and secondary contact. The second was that lineage diversification was driven by a longer term geographic barrier while admixture was facilitated by human movement across the African continent during the Holocene era. Here, we find evidence for shared aspects of population history and expansion across *Ae. lilii*,* Ae. bromeliae*,* Ae. africanus,* and *Ae. aegypti,* even though only *Ae. aegypti*, and to some extent *Ae. bromeliae,* have a close association with humans. This supports the first hypothesis that a common historical event, such as past climate change, shaped their evolutionary past. *Aedes. hansfordi* is inconclusive since although the significant Tajima's *D* values support population expansion, divergent mtDNA haplogroups were not detected as in the other species. However, the latter may be due to limited sample size. Greater sampling from its broad distribution across sub‐Saharan Africa (Huang, 1997) is needed to determine whether *Ae. hansfordi* also exhibits historical divergence and admixture.

The ABC analyses for *Ae. bromeliae* and *Ae. lilii,* significant *F*_ST_ values and the mtDNA haplotype networks for these species, as well as for *Ae. africanus* collectively present evidence for historical allopatric divergence. Within *Ae. bromeliae* and *Ae. lilii*, ABC analysis of the nuclear loci for both species and of the mitochondrial *COI* gene for *Ae. lilii* was found to fit the same demographic model as that previously identified for African *Ae. aegypti* (Bennett et al., 2016). The most probable model choice and posterior estimates suggest this divergence occurred in multiple forest refugia during the late Pleistocene between 8,000 and 116,000 years ago, in accordance with the most recent estimated glacial periods (Castañeda et al., 2009; Gasse, 2000) (Table S3). Although the ABC model choice for the mitochondrial *COI* gene indicates a contrasting demographic history for *Ae. bromeliae* not involving admixture, it still supports geographical divergence throughout the late Pleistocene and Holocene. The mtDNA haplotype networks of *Ae. africanus*,* Ae. lilii,* and *Ae. bromeliae* show a particularly clear signal of historical divergence in allopatry with at least two highly divergent clusters of haplotypes in each species. This signal is likely more clearly seen in the mitochondrial rather than the nuclear genes due to the smaller effective population size of mtDNA, which makes it more sensitive to genetic drift coupled with the lack of recombination following admixture (Ballard & Whitlock, 2004; Hare & Avise, 1998; Toews & Brelsford, 2012). As a result, mtDNA is often more structured than nuclear DNA after population isolation and secondary contact, as reported in other studies (Berggren, Ellegren, Hewitt, & Seddon, 2005; Eggert, Rasner, & Woodruff, 2002; Franck et al., 2001; Janko et al., 2007; Monsen & Blouin, 2003; Yang & Kenagy, 2009). The particularly high‐mtDNA divergence of 5.8% in *Ae. africanus* is indicative of allopatric fragmentation having led to speciation (Chan et al., 2014; Cywinska, Hunter, & Hebert, 2006; Ruiz‐Lopez et al., 2012). The lack of reciprocal monophyly at nuclear loci does not concur with this, but this could be due to the greater time required for lineage sorting in nuclear loci. The presence of cryptic species within *Ae. africanus* will be explored elsewhere using additional loci.

The large extent of forests during the early Holocene could have facilitated range expansion and provided opportunities for gene flow between populations, which were previously restricted by unsuitable habitat (Haffer, 1997, 2008; Hewitt, 1996). We report evidence for such expansion in all species excepting *Ae. africanus*, with significant values of Tajima's *D* at one or more nuclear genes for African species. In addition, numerous significant values of Tajima's *D* and Fu's Fs are seen within regional groups for all species. In addition to this, an increase in population size was revealed by GMRF skyride for haplogroup 2 of mitochondrial *COI* for both *Ae. lilii* and *Ae. africanus*. However, Bayesian skyline analysis was unable to detect signals of expansion in other species and molecular datasets. This could be because admixture, which we have detected in all African populations, homogenizes genetic variation and so acts to remove signals of demographic history such as a change in effective population size (Brown, Jordan, et al., 2011; Qu et al., 2011, 2012; Sonsthagen, Chesser, Bell, & Dove, 2012). Although it is known that population structure can impact on Bayesian skyline methods (Heller, Chikhi, & Siegismund, 2013), biasing and confounding factors have not been fully assessed (Gattepaille, Jakobsson, & Blum, 2013).

Admixture was detected by ABC for both *Ae. bromeliae* and *Ae. lilii* using the nuclear loci and can be visualized as the mixing of genetically diverse haplotypes across geographically disparate populations in the haplotype networks of nuclear *IDH2* and *RpL30b* for both these species and that of *Ae. africanus*. Additional evidence for admixture is provided by the *COI* haplotype network for *Ae. lilii,* in which variants from both divergent haplogroups are present in all geographic regions. This is similar to the situation found in *Ae. aegypti* previously, attributed to range contraction and expansion in response to cyclic climate change (Bennett et al., 2016).

Faunal comparisons of regional forests within Africa have shown differences in the distribution of taxa; while many species are shared between the West and Central African rainforests (Guineo‐Congolian), few are shared between these and the coastal forests of East Africa and the Eastern Arc mountain ranges (Couvreur et al., 2008; Kadu et al., 2013; Wagner et al., 2008). From this, it has been suggested that the East African forests have remained more isolated throughout history than have the West and Central African rainforest blocks. In support of this, we find evidence of strong, genetic population structure between populations of *Ae. bromeliae* in Tanzania, East Africa and Uganda, Central Africa through elevated F_ST_ values for all genetic loci and geographic structure in the haplotype network of mitochondrial *COI*. By contrast, in *Ae. lilii,* the haplotype networks for all loci revealed little to no genetic structure between Benin, West Africa and Uganda, Central Africa and although F_ST_ values indicate a significant difference at mitochondrial *COI* this is lower than that found in *Ae. bromeliae* between Tanzania, East Africa and Uganda, Central Africa. A corresponding pattern of greater East‐Central than West‐Central differentiation is not readily evident in *Ae. aegypti,* but we note that significant population structure was found at three loci for the former comparison while at only two for the latter. Rather than relating to differences in forest connectivity per se, greater East‐Central African differentiation could result from another potential geographical barrier which restricts mosquito dispersal between East and Central Africa, that is, the East African Rift (Minakawa, Mbogo, & Yan, 2003; Braginets et al. 2000; Lehmann et al., 1999, 2000, 2003).

In relation to this, it has been suggested that cyclic isolation of the east coast and Eastern Arc mountain forests from the Guineo‐Congolian block may be one factor explaining the high levels of endemicity in East Africa (Couvreur et al., 2008; Voelker, Outlaw, & Bowie, 2010); while periodic connections allowed taxa to move between forests, subsequent contraction of forest habitat leads to vicariance events. Studies have generally provided evidence for ancient connections between the east and Guineo‐Congolian rainforests with divergence between regional taxa dating to climate events within the Miocene or Plio‐Pleistocene (Bowie et al., 2006; Burgess, Clarke, & Rodgers, 1998; Kadu et al., 2013; Matthee et al., 2004; Measey & Tolley, 2011) or signals of expansion and secondary contact between species complexes (Bowie et al., 2004) dating to the Pleistocene. For example, divergence dates between *Annonaceae* flowering plant species of the Guineo‐Congolian and East African forests reveal multiple origins of this rainforest restricted taxon throughout the last 8–33 million years (Couvreur et al., 2008). This raises the possibility that climate‐related vicariance could have been important in the speciation of *Aede*s mosquitoes, including within the Simpsoni Complex, whose geographical distributions are currently divided across the African continent (Bennett et al., 2015). The signal of admixture which we have detected within *Ae. bromeliae* from Tanzania and Uganda and *Ae. lilii* from Benin and Uganda using ABC analysis suggests a more recent connection of forest habitats across the African continent. This is in keeping with pollen and soil data suggesting that forest cover was at its maximum during the early Holocene era beginning 11,000 years ago (Castañeda et al., 2009; Lovett & Wasser, 2008; Olago, 2001; Watrin, Lézine, & Hély, 2009). Therefore, climate‐induced habitat change is likely to have impacted on *Aedes* mosquitoes throughout their evolutionary history, generating both inter‐ and intraspecific genetic diversity.

## 5 CONCLUSION

The genetic variation observed within and between *Aedes* species in this and previous studies (Bennett et al., 2016) advocate for east‐west differences in population structure across Africa. Interestingly, arboviruses such as CHIK and YFV also have East and West African lineages (Mutebi, Wang, Li, Bryant, & Barrett, 2001; Powers, Brault, Tesh, & Weaver, 2000), which could be promoted by restricted gene flow between both ancestral and modern vector populations, which we report here. Viruses will be under selection to adapt to specific local invertebrate hosts which can enhance vector competence and so disease transmission (Weaver & Reisen, 2010). Geographic differences in the distribution of vector genotypes and species are therefore likely, at least partly, to influence disease susceptibility and therefore arboviral disease transmission across Africa.

In this and previous studies (Bennett et al., 2016), *Aedes* species are characterized by the admixture of genetically divergent lineages and exhibit high effective population sizes. This high level of genetic diversity indicates a high adaptive potential (Facon et al. 2006; Rius & Darling 2014) which may be beneficial for endurance during climate extremes and adaptability to peridomestic environments. This, together with increased contact of forest mosquitoes with the developing human population, raises concern over whether other *Aedes* mosquitoes will follow in the evolutionary tracks of *Ae. aegypti* and advance into domestic environments, increasing arboviral disease transmission in the future.

## CONFLICT OF INTEREST

None declared.

## DATA ACCESSIBILITY

DNA sequences: GenBank Accession nos MF183226–MF183441 and MF183443–MF183893. Sampling locations and/or online‐only appendices uploaded as online supplemental material.

## AUTHOR CONTRIBUTIONS

C.W. and K.L.B. designed the study. K.L.B., M.K, F.S., R.D., G.M., J.L., Y‐M.L., and C.W. carried out the fieldwork and contributed samples. K.L.B. performed the research and analyzed the data under the supervision of C.W. Y.‐M.L. contributed to sequencing of the *COI* gene. K.L.B. wrote the manuscript with help from C.W. All authors contributed to comments and approved the final version.
