# Supplementary material for: Comparative phylogeography of Aedes mosquitoes and the role of past climatic change for evolution within Africa
Source: Ecol Evol. 2018 Feb 16;8(5):3019–36. doi: 10.1002/ece3.3668 (PMC5838080; doi:10.1002/ece3.3668)
Supplement: Supplementary file 1 [file ECE3-8-3019-s001.pdf]

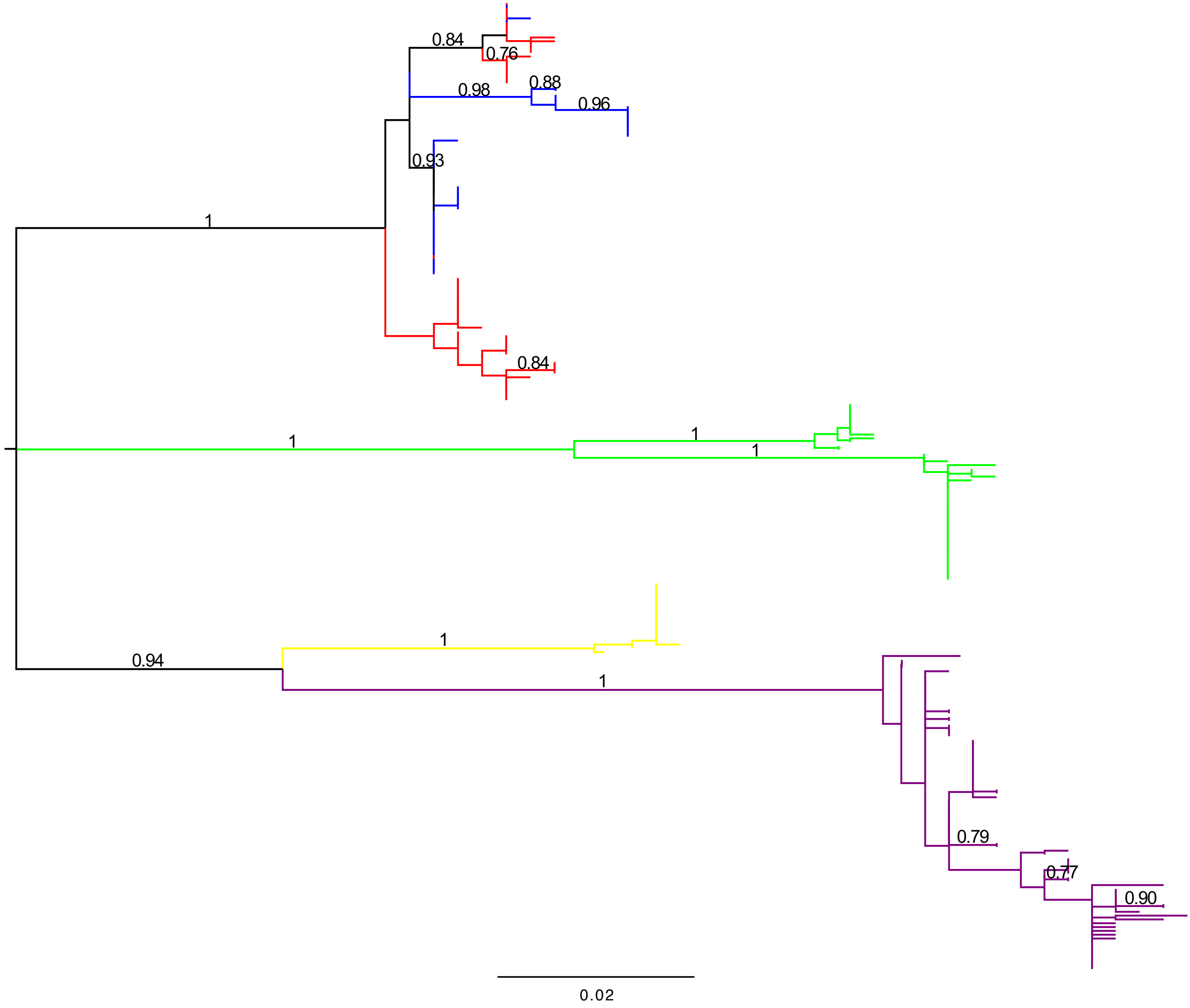

Figure S1. The maximum likelihood tree for mitochondrial *COI* created in MEGA 7 using the best available substitution model as chosen by jModelTest, including sequences from *Ae. bromeliae* (red), *Ae. lilii* (blue), *Ae. africanus* (green), *Ae. hansfordi* (yellow) and *Ae. aegypti* (purple). The substitution model was Tamura & Nei with invariant and gamma distributed rates among sites. The tree was constructed from 1000 bootstraps; values over 0.7 are shown.
